# A Mobile Analytical Device for On-Site Quantitation of Anthocyanins in Fruit Beverages

**DOI:** 10.3390/mi12030246

**Published:** 2021-02-28

**Authors:** Mohsen Salimi, Brigitta R. Sun, Jenny Syl Tabunag, Jianxiong Li, Hua-Zhong Yu

**Affiliations:** 1 Department of Chemistry, Simon Fraser University, Burnaby, BC V5A 1S6, Canada; mo_salimi@chem.iust.ac.ir (M.S.); rsa100@sfu.ca (B.R.S.); jstabunag@protonmail.com (J.S.T.); 2 Faculty of Chemistry, Iran University of Science and Technology, Tehran 16846-11367, Iran; 3 Laca Biotech Inc., 120-3771 Jacombs Rd., Richmond, BC V6V 2L9, Canada; jli@sfu.ca

**Keywords:** anthocyanin, Wi-Fi scanner, mobile app, colorimetry, on-site detection

## Abstract

Anthocyanins are antioxidant and anti-inflammatory ingredients in various fruit beverages, for which their conservation and quantitation are important for the food industry. In this paper, we report a simple, portable device for accurate on-site determination of total monomeric anthocyanins in fruit beverages employing a Wi-Fi scanner coupled with a flexible microchip and a free mobile app. The detection principle is based on the pH-induced colorimetric reactions of anthocyanins performed in a specially designed microchip and validated with standard spectrophotometric measurements. The microchip with multiple testing vials was prepared with the benchtop molding method with a common PDMS elastomer and a transparency film; the photo of the scanned microchip is wirelessly sent to a smartphone and the RGB values of individual reaction vials in the microchip are analyzed with a free mobile app to determine the corresponding concentrations. It was demonstrated that the quantitation performance of this POCT device is comparable with conventional spectrophotometry in the determination of total anthocyanins in both standard solutions and fruit beverages.

## 1. Introduction

Anthocyanins are a large group of phenolic pigments, which exist in various fruits, vegetables, and grains [1,2,3]. They contribute to the color and appearance of many root vegetables and fruits such as blueberries, raspberries, strawberries, cherries, and purple grapes. There are over 600 derivatives of anthocyanins in nature, and different plant species have varied types and amounts of anthocyanins [1,2,3]. According to Basu et al. [4], anthocyanins can decrease the amount of low-density lipoprotein, plasma glucose, and total cholesterol, which are effective in preventing coronary artery diseases. Faria et al. reported that blueberry anthocyanins can inhibit cancer cell proliferation by acting as antiinvasive factors and chemo-inhibitors [5]. The assurance of the richness in total anthocyanins for the determination of the proper harvest time of fruits and the quality control of beverage production (from non-acholic juices to red wines) in breweries is essential [6,7]. It is worth mentioning that the proper fermentation process is important not only for wine brewing, but also for other beverage products such as fruit beers (as reported recently by Salanța et al. [8]).

The most common methods for the determination and quantitation of anthocyanins are UV–VIS absorption spectrophotometry and high-performance liquid chromatography (HPLC). Since it is rapid and simple, the former has been widely applied for the quantitation of total anthocyanins in laboratories and food industries. This method was first introduced by Sondheimer and Kertesz [9] in 1948, which was officially adopted by the Association of Official Agricultural Chemists (AOAC) in 2005 [10]. It is often performed by first adjusting the pH value, followed by spectrophotometric analysis. In recent years, HPLC has also been commonly used as a reliable method for total and individual anthocyanins measurements, for which the quantitation can be achieved by using an external standard (e.g., cyanidin-3-glucoside). However, it remains as a central laboratory method due to the high cost of the instrumentation and the required training of operators [2,6,10,11,12,13]. On the other hand, isolated anthocyanins are highly unstable and decay easily [14]. Thus, there is a practical need for a rapid, user-friendly system for the on-site quantitation of total anthocyanins in commercial products.

On-site chemical detection and point-of-care diagnosis have been appealing to both academia and industry (not limited to biomedical diagnostics) in the past decades because of their capability of providing low-cost and user-friendly analytical tools for measurements that are remote from central laboratories [15,16,17,18]. There have been a few attempts to develop portable systems for anthocyanin quantitation and almost all of them have been based on infrared spectroscopy [7,11,19]. For example, Baca-Bocanegra et al. used a portable micro-NIR spectrophotometer (908–1676 nm) to determine the phenolic compounds of red grapes by directly taking spectra of intact grapes and grape skins [20]. These methods are nondestructive, and their responses are fast; however, the instrumentation is rather expensive, the operation is still complex, and the data may not be as accurate as the traditional HPLC methods.

Smartphones today operate as miniature computers with superior data transmission and photo taking capabilities; they are affordable even in less developed countries and most people are familiar with their function. Due to these advantages, there has been growing interests in adapting smartphones for on-site chemical analysis and point-of-care testing [15,21,22,23,24]. Nonetheless, smartphones suffer in the accuracy of quantitative analysis because of nonuniform and non-reproducible lighting, particularly for optical imaging applications [15,25,26]. For solving these problems, researchers have tried different strategies of using either 3D-printed optical accessories [27], or external flat light sources [28], in order to provide uniform illumination for imaging. Alternatively, Meng et al. used an office scanner for taking high-quality images of colorimetric assays for organophosphorus pesticides in food products [29]; while the approach indeed improved the accuracy and reproducibility of the tests, the need for stand-alone computer and imaging processing software impedes the “true” portability of such a device for on-site chemical analysis.

Herein, we describe a simple on-site detection device using a smartphone/tablet and a portable scanner that are wirelessly connected to each other for the precise determination of the total anthocyanins in fruit beverages. Reusable PDMS-based microchips are designed to fit the dimension of the scanner for performing the colorimetric reactions, the assay images are wirelessly sent to a smartphone, and the analysis is performed using the pre-installed, free Color Grab app. It is not only convenient for the establishment of calibration curves, but also for testing multiple samples in a single experiment.

## 2. Materials and Methods

### 2.1. Reagents and Materials

Potassium chloride (Mallinckrodt Pharmaceuticals, Bedminster Township, NJ, USA), Sodium acetate (Caledon Laboratory Ltd., Georgetown, ON, USA), Polydimethylsiloxane (PDMS) fabrication kit (Dow Chemical Co., Midland, MI, USA), Transparency film (ACCO Brands, Lake Zurich, IL, USA), alumina powder (65–325 mesh, Fisher Chemicals, Hampton, NH, USA), HCl (37% ULSI grade, Gem Chemicals, Evansville, IN, USA), and cyanidin-3-O-β-glucoside chloride (Cy3G) standard (98% HPLC purity, Aobious Inc., Gloucester, MA, USA) were used as received unless otherwise noted. All the beverage samples were provided by Laca Biotech Inc. and tested as received (except for dilution when needed). Deionized water (>18.2 MΩ·cm) used throughout all experiments to prepare standard solutions and samples was produced freshly with a Barnstead EasyPure UV/UF compact water system (Dubuque, IA, USA).

### 2.2. Sample Preparation and UV–Visible Spectrophotometric Analysis

A 140-μg/mL stock solution of the anthocyanin standard (Cy3G) was prepared and diluted with two different buffers (pH 1.0 and pH 4.5) to a range of concentrations from 1.0 to 28 μg/mL. The pH 1.0 “buffer” was prepared with KCl and HCl [10]; the pH 4.5 buffer was prepared with sodium acetate and HCl (1.0 M). The dilution factor was kept constant as 1/5 (one part of the stock solution and 4 parts of buffer) so that it would not exceed the buffer capacity.

An Ocean Optics QE65000 UV–VIS spectrophotometer equipped with a DH-2000-BAL deuterium and tungsten light source and a 600-μm SR optical fiber probe (Winter Park, FL, USA) was employed for the spectrophotometric analysis. The spectra were recorded using the manufacturer-provided software over a wavelength range of 340–800 nm in a 1.0 cm pathlength Kartell Visible Range cuvette (HCS Scientific & Chemical Pte Ltd., Singapore).

### 2.3. Device Fabrication and Mobile Colorimetric Analysis

**Scheme 1:**
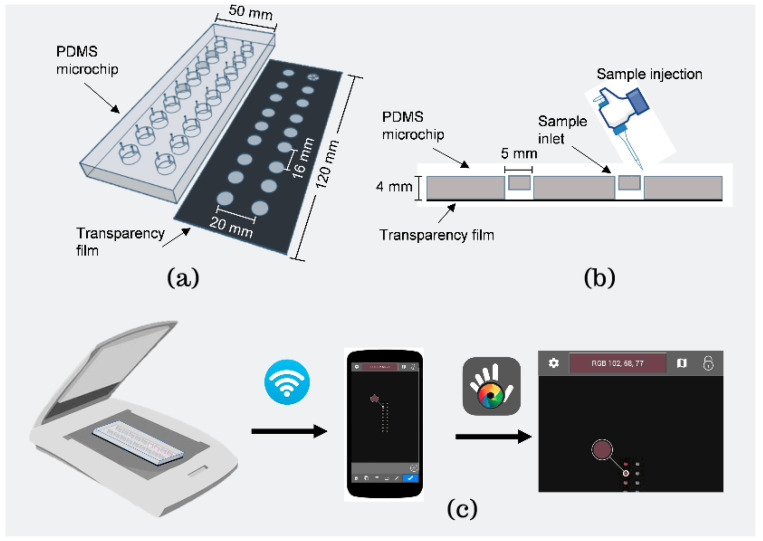
Schematic illustration of the “mobile” analytical device employing a PDMS chip and a Wi-Fi-enabled portable scanner. (**a**) Fabrication of the device with a PDMS chip and a matching transparency film; the PDMS chip with two rows of mini-vials was affixed on the transparency film that was painted in black (except for the areas matching the bottom of reaction vails). (**b**) A cross-section view of the device showing the chip configuration and how the sample is added. (**c**) Imaging the PDMS chip with a WiFi-enabled scanner and wirelessly sending photos to a smartphone for data processing using the Color Grab app.

As shown in Scheme 1a, the device for performing mobile colorimetric analysis consisted of a PDMS plate with 18 mini-vials (with openings at the top for solution injection) and a matching base (a transparency film that was printed black except for the 18 circular sections facing each of the mini-vials, vide infra). The preparation of the PDMS microchip using the standard commercial kit, a set of mini-cylinders, and a set of mini-magnets is presented in the Supplementary Materials (Scheme S1). The assembly of the device and dimensions of the reaction vails are illustrated in the cross-section view shown in Scheme 1b. A Doxie Flip™ cordless flatbed scanner with an ez Sh@re WiFi SD Memory Card Adapter was used for scanning the microchip and sending photos to the smartphone/computer wirelessly (Scheme 1c). A smartphone (Samsung galaxy S3) with the installed Color Grab app was employed for the quantitative analysis of the assay results. The images were scanned with the highest resolution (600 dpi) and saved in JPEG format.

## 3. Result and Discussion

### 3.1. Detection Principle and Spectrophotometric Calibration

**Figure 1:**
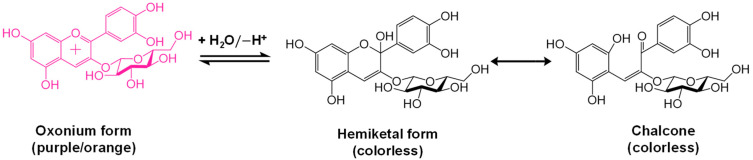
pH-dependent structural conversion of anthocyanin from purple-colored flavylium cation (dominant at pH 1.0) to the two resonant structures (hemiketal/chalcone) that are colorless (dominant at pH 4.5).

As mentioned above, the AOAC official method 2005.02 established by Lee et al. [10] has been commonly adapted as the standard protocol for the determination of total monomeric anthocyanins in fruit juice, beverages, natural colorants, and wines. The detection principle is based on the pH-dependent structural conversion of anthocyanins [2,10]. As shown in Figure 1, anthocyanins (represented with Cy3G) shift from the purple/orange-colored flavylium cation (dominant at low pH) to a pair of colorless, resonant structures (hemiketal and chalcone that become dominant species at pH 4.5) via hydration followed by proton loss [10]. As reported by Tang et al. [30], such a pH-dependent structural switching is reversible, which was also confirmed with a raw juice sample in this study (presented in Supplementary Materials, Figure S1).

**Figure 2:**
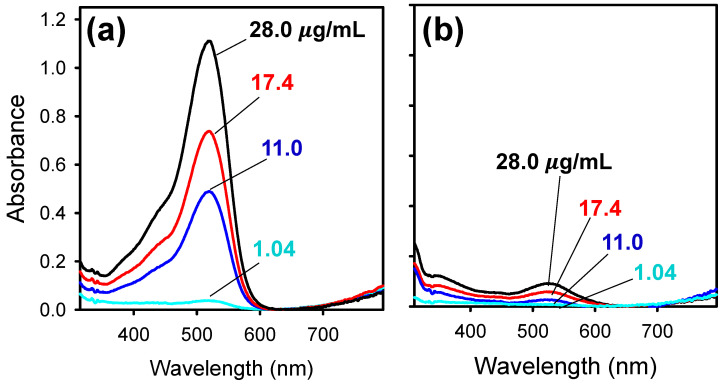
Representative UV–VIS absorption spectra of anthocyanin (Cy3G) standards of different concentrations (as listed) at (**a**) pH 1.0 and (**b**) pH 4.5.

In Figure 2, we show the UV–VIS spectra of Cy3G standard solutions at varied concentrations; in particular, we obtained the spectra of each of the standard solutions at both pH 1.0 (Figure 2a) and pH 4.5 (Figure 2b) for a direct comparison. The strong band centered at 520 nm in the visible region corresponds to the fully delocalized π-conjugated system of flavylium cation (Figure 1), which is disrupted by the hydration process (reacting with a water molecule at the 2-position followed by releasing a proton); the clear contrast of the absorbance at two different pH values confirmed the dominant species are different. It is also apparent that the higher the concentration of Cy3G, the stronger the spectrum becomes. At pH 1.0, with as low as 1.04 μg/mL of Cy3G, we can observe a clearly defined absorption peak that would form the basis for the subsequent calibration and quantitation.

**Figure 3:**
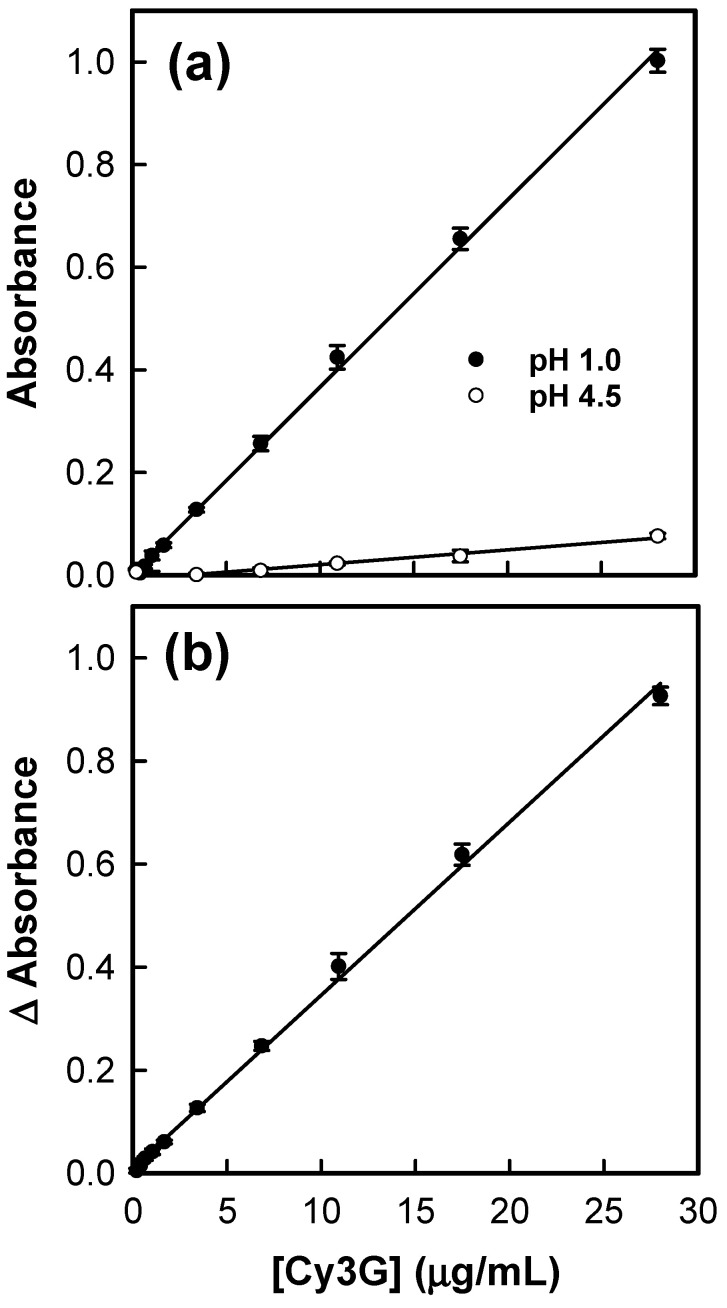
(**a**) Absorbance at different concentrations of Cy3G at pH 1.0 (solid circles) and pH 4.5 (open circles). (**b**) Dependence of the absorbance difference (∆A = A_(pH 1.0)_ − A_(pH 4.5)_) as a function of the Cy3G concentration. The solid line shows the best linear fit to the experimental data.

The absorbances of the full set of Cy3G standard solutions at two different pH values are plotted as a function of their respective concentrations in Figure 3a; in both cases, the measured absorbance values are reproducible as indicated by rather small error bars (Table S1), though the absorbances differ from each other over an order of magnitude. For example, the absorbance for the highest concentration standard (28 μg/mL) is 1.10 at pH 1.0 but reduces to 0.11 at pH 4.5. The results shown in Figure 3a also indicate that the absorbance at pH 4.5 cannot, in fact, be simply omitted; therefore, it is more reasonable to build the calibration curve using the difference in the absorbances, i.e., ΔA = A_(pH 1.0)_ − A_(pH 4.5)_. In retrospect, such a differential approach was adapted by Lee et al. in the initially proposed method [10], and thus established calibration curve indeed showed excellent linearity (Figure 3b). The best linear fit to the experimentally determined ΔA vs. [Cy3G] data yields a close-to-unity R^2^ value (0.998) and a correlation equation of ΔA = 3.4 × 10^−2^ [Cy3G] + 1.0 × 10^−2^. The determined limit of detection (LOD) and limit of quantitation (LOQ) are 0.56 and 1.9 μg/mL, respectively, based on the 3*σ*_b_/*k* and 10*σ*_b_/*k* values (where *k* is the slope and *σ*_b_ is the standard deviation of the y-intercept of the regression line).

### 3.2. Chip Design and Mobile Colorimetric Analysis

In our previous work [29], we fabricated a transparent microchip for the quantitation of pesticides that consists of a PDMS cover, a PDMS channel plate, and a polycarbonate (PC) base. The current microchip design shown in Scheme 1a implemented the following improvements: (1) the PDMS microchip as prepared via the “mobile” mini-cylinder approach (Supplementary Materials, Scheme S1) offers the flexibility in changing the position and number of reaction vials; (2) the addition of alumina powder in PDMS precursors makes the microchip opaque that helps to reduce the interference from the off-vial lights; (3) the use of a black-printed transparency film instead of a PC plate further improves the contrast for scanning and imaging.

**Figure 4:**
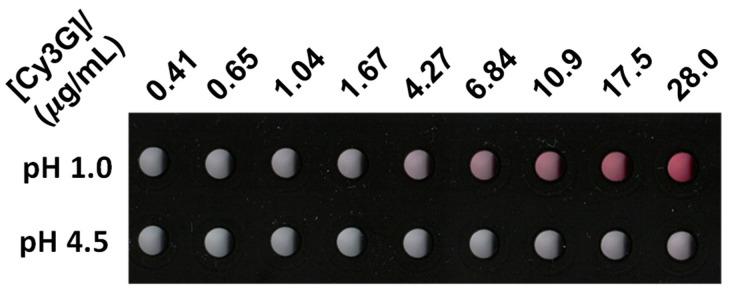
A scanned photo of the PDMS chip where the colorimetric reaction occurred in the two set of mini-vails.

Digital image analysis has been extensively used for quantifying colorimetric assays, which is typically performed by using a smartphone, digital camera, or scanner to capture the image and process the color information [31]. As such, all mini-vails on the microchip were essentially adapted for performing the colorimetric assays; as shown in Figure 4, the two rows of assay vials with different concentrations of Cy3G showed varied colors, from plain gray to bright pink in the top row (pH 1.0), with essentially no clear color change in the bottom row (pH 4.5).

**Figure 5:**
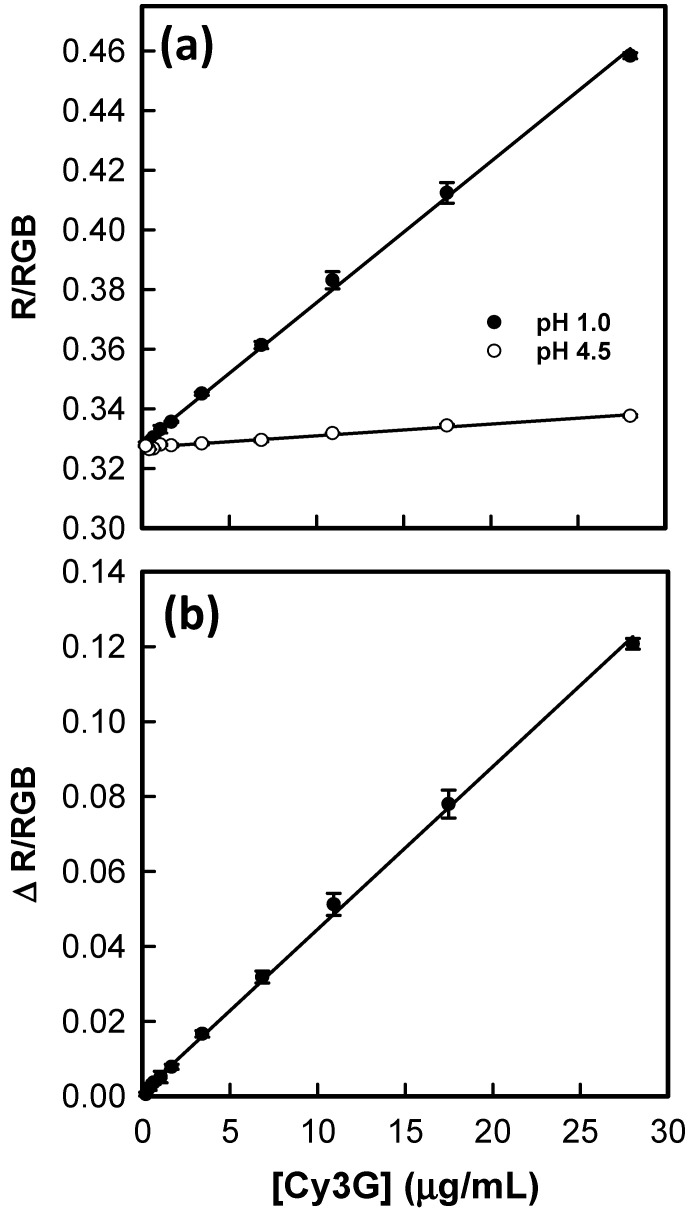
(**a**) R/RGB value at different concentrations of Cy3G at pH 1.0 (solid circles) and pH 4.5 (open circles). (**b**) ∆(R/RGB) (difference between the values at pH 1.0 and pH 4.5) vs. the Cy3G concentration, where the solid line shows the best linear fit to the experimental data.

While a number of different algorisms exist, the RGB (red, green, and blue) color space is the most popular approach to describe a color quantitatively [29,31,32]. As presented in the Supplementary Materials (Figure S2), we have shown that the normalized R value (R/RGB) is the best choice for analyzing the colorimetric assay for anthocyanins on the PDMS chip. The RGB value refers to the sum of all three channels (R, G, B) obtained for the same assay vail (R + G + B). As depicted in Figure 5a, we have shown that the R/RGB value increases substantially with the increased concentration of Cy3G at pH 1.0, a phenomenon that was not clearly observed at pH 4.5 (while discernible increases are still evident). Three independently prepared assays (Supplementary Materials, Figure S3) were performed to demonstrate the reproducibility of the colorimetric method and were used to calculate the standard deviations of the data (shown as error bars) in Figure 5a. In line with the spectrophotometric analysis, we established the calibration curve by plotting the difference in R/RGB values between those of pH 1.0 and pH 4.5 as a function of the Cy3G concentration (Figure 5b). The linear regression line has an R^2^ value of 0.999, and the correlation equation, ΔR/RGB = 4.3 × 10^−3^ [Cy3G] + 1.2 × 10^−3^, has been deduced as well. It is remarkable that the determined LOD (0.40 μg/mL) and LOQ (1.3 μg/mL) values are comparable (slightly improved, as a matter of fact) with those of spectrophotometric analysis described above.

### 3.3. Sample Measurements and Validation with the Standard Method

Due to the complexity of anthocyanin pigments (varied derivatives and abundances), the anthocyanin content of real-world samples is usually calculated as the content of an anthocyanin standard material that is the major species (Supplementary Materials, Table S2). For the conventional spectrophotometric analysis, it was suggested that the concentration of total anthocyanins is calculated as an equivalent of Cy3G, which is, nonetheless, the major anthocyanin species in nature [2]. We adapted the same approach to quantitate the total anthocyanins in a number of beverage samples, i.e., to determine the equivalent concentration of Cy3G using the microchip device. In particular, the samples included four raw grape juice beverages and five fermented wines (Supplementary Materials, Figure S4). All samples were obtained from the production line of the Bayou Brewing Club, which is a sub-division of Laca Biotech Inc., Richmond, BC, Canada.

**Figure 6:**
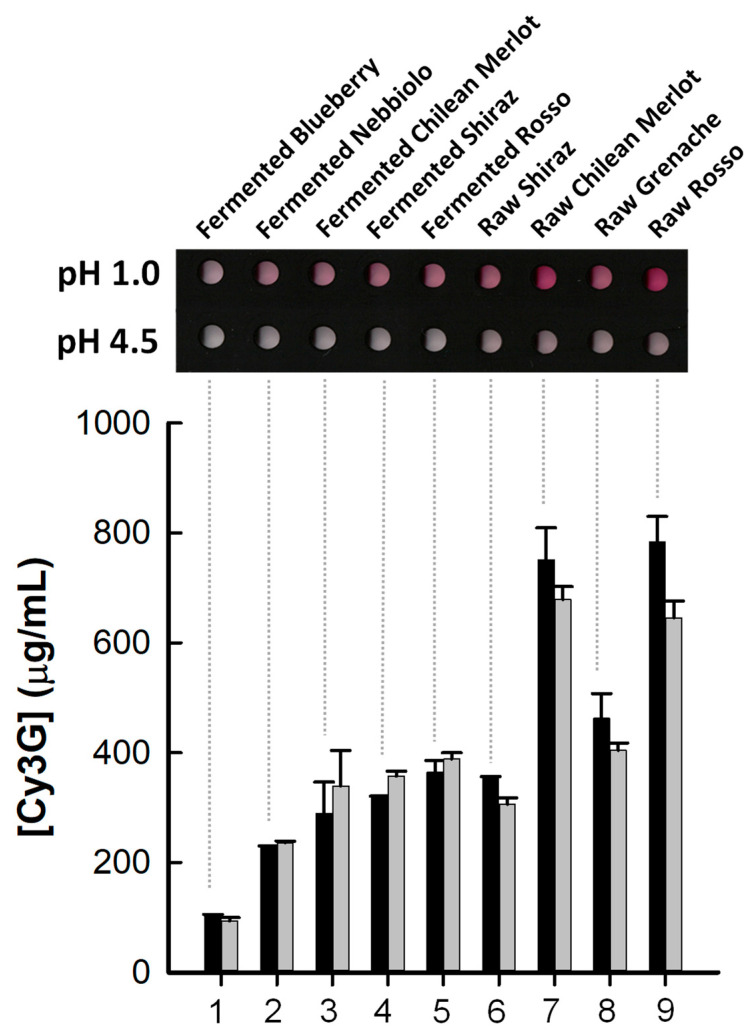
Comparison of the standard spectrophotometric (gray bars) and microchip (black bars) methods in the determination of total anthocyanins in fruit beverages. The top inset is the scanned photo of the PDMS chip employed for the analysis.

As shown in Figure 6 (top inset photo), all beverage samples displayed a clear difference in the apparent color upon changing pH from 1.0 (top row) to 4.5 (bottom row). With the aid of the calibration curves established above, the concentrations of Cy3G in all these samples were determined and plotted together for direct comparison (Figure 6). The standard deviations were obtained from three repeated measurements (Supplementary Material, Figure S5). With only a few exemptions, the spectrophotometric and microchip determined concentrations (denoted as [Cy3G]_Abs_ and [Cy3G]_Mic_, respectively) are in good agreement (i.e., within the experimental uncertainties). It is also noticeable that the fermented samples (wines) typically have less anthocyanins in comparison with the raw beverages (juices), which can be attributed to the degradation of anthocyanins under elevated temperature (35 °C) and in the presence of yeasts for a prolonged time period (two weeks).

**Figure 7:**
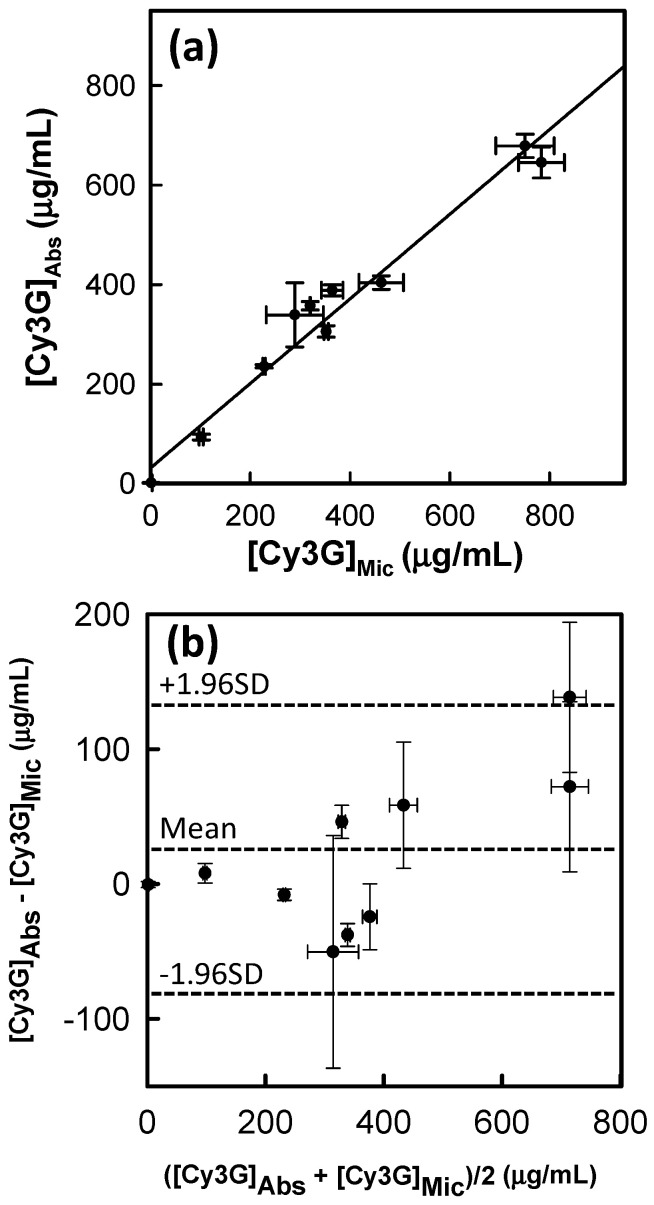
(**a**) Correlation between the concentration values measured via the mobile microchip and spectrophotometric method, denoted as [Cy3G]_Abs_ and [Cy3G]_Mic_, respectively. (**b**) The Bland–Altman plot demonstrates the agreement between the two methods.

For a better comparison of the two methods, the determined concentrations of all samples ([Cy3G]_Abs_ vs. [Cy3G]_Mic_) are plotted in Figure 7a; the satisfactory linearity (R^2^ = 0.970) and close-to-unity slope (0.85 ± 0.05) of the regression line confirm the correlation between the two quantitation methods. A less important observation is that the experimental uncertainties (error bars) are mostly close to each for each of the samples, indicating that the sample preparation process is the main source of experimental errors for both methods. The Bland–Altman plot shown in Figure 7b further validates the applicability of the mobile microchip method in the concentration determination of the total anthocyanins, as all (but one) experimental data fall in the ±1.96SD range [33].

It should be noted that the determination of total anthocyanins in fruit beverages is merely a showcase example of real-world applications of the “mobile” analytical device proposed herein. The ultimate motivation was to perform on-site quality control tests with a portable scanner and reusable microchip; more accurate determination or separation of different anthocyanins shall still rely on laboratory-based techniques, such as HPLC and MS that have much higher precision and better detection limits [2,6]. Nonetheless, the reported “mobile” analytical device, by integrating a portable scanner (Doxie & Co. LLC, Raleigh, NC, USA; MSRP USD 149), a reusable microchip, and a free color analysis app, indeed promises a low-cost, simple, and quantitative method for screening the anthocyanin abundance of a wide range of real samples, from juices and wines to fruit beers, which can be extended to many other industrial products and processes. In addition, the same device design can be potentially adapted for point-of-care testing of disease markers that are essential for resource-limited settings or remote locations.

## 4. Conclusions

In this study, a portable scanner coupled with a smartphone app was adapted for on-site quantitation of anthocyanins in fruit beverages. For this aim, a colorimetric assay was performed on a specially designed PDMS microchip; the images were wirelessly transmitted to a smartphone and subsequently analyzed with the free Color Grab app. We have shown the direct correlation of this microchip method and the standard spectrophotometric technique, in that both are based on pH-induced structural change among different forms of anthocyanins. Besides the established calibration curves in both measurements, we have shown the capability of the mobile device for the screening of total anthocyanins in a number of raw and fermented fruit beverages. This integrated, mobile device promises a powerful analytical tool for rapid, low-cost, on-site measurements where standard colorimetric assays are available.

## Data Availability

The data presented in this study are available in the article and on-line Supplementary Materials.

## Supplementary Materials

The following are available online at https://www.mdpi.com/2072-666X/12/3/246/s1, Detailed procedure to prepare the PDMS microchip and additional experimental data (images) for the colorimetric determination of anthocyanins in both standard solutions and beverage samples. Scheme S1: Detailed procedure to prepare the PDMS microchip; Figure S1: Reversibility test of the pH dependent structural change of anthocyanidins; Table S1: Three replicate UV-Vis measurements of Cy3G standard solutions; Figure S2: R, G, and B intensities of Cy3G standard solutions at different concentrations; Figure S3: Three repeated experiments to determine the colorimetric response of Cy3G standard solutions with the microchip device; Table S2: Six naturally occurring anthocyanidins and their corresponding abundance; Figure S4: Tested beverage samples and their natural colors.; Figure S5: Three replicate experiments for determining the concentrations of Cy3G in different beverage samples with the microchip device.
