# Supplementary material for: A Mobile Analytical Device for On-Site Quantitation of Anthocyanins in Fruit Beverages
Source: Micromachines (Basel). 2021 Feb 28;12(3):246. doi: 10.3390/mi12030246 (PMC7997336; doi:10.3390/mi12030246)
Supplement: Supplementary file 1 [file micromachines-12-00246-s001.pdf]

Supplementary Materials

A Mobile Analytical Device for On-Site Quantitation of Anthocyanins in Fruit Beverages

Mohsen Salimi,[1,2] Brigitta R. Sun,[1] Jenny Syl Tabunag,[1] Jianxiong Li,[3] and Hua-Zhong Yu[1,*]

[1] Department of Chemistry, Simon Fraser University, Burnaby, BC V5A 1S6, Canada

[2] Faculty of Chemistry, Iran University of Science and Technology, Tehran 16846-11367, Iran

[3] Laca Biotech Inc., 120-3771 Jacombs Rd., Richmond, BC V6V 2L9, Canada

* Corresponding author; e-mail: hogan_yu@sfu.ca

Detailed procedure to prepare the PDMS microchip and additional experimental data for the spectrophotometric and colorimetric determination of anthocyanins in both standard solutions and beverage samples.

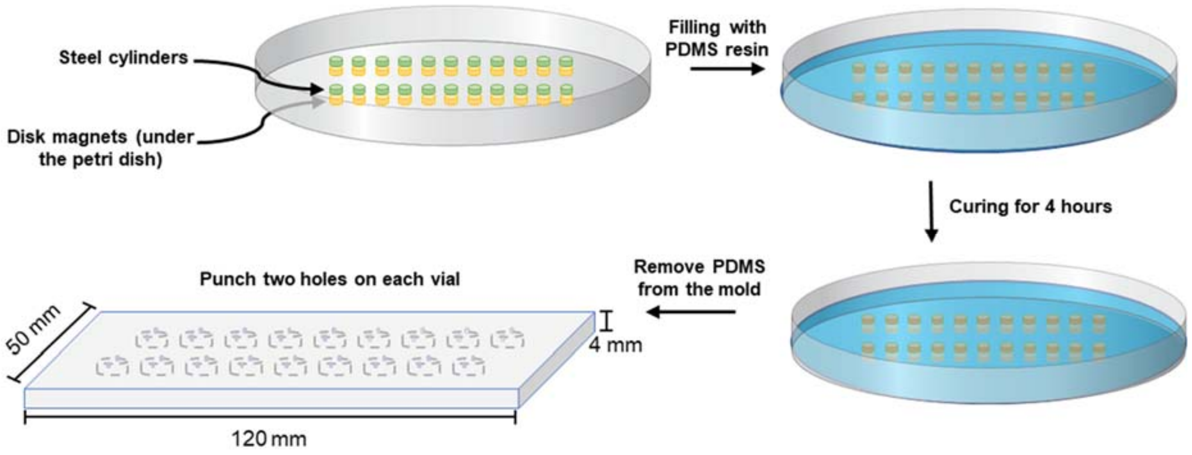

Scheme S1. Detailed procedure to prepare the PDMS microchip.

Firstly, 18 stainless steel cylinders (5 mm in diameter and 3 mm thick) were placed in a petri dish (16 mm apart from each other in two rows), and "immobilized" by attaching disc magnets (5 mm in diameter and 3 mm thick) underneath the petri dish. Subsequently, the petri dish was filled with PDMS precursors; particularly the silicone elastomer base and the curing agent were mixed at 10:1 ratio with 4 % (w) alumina powder added. The mold was placed in a vacuum chamber for degassing for one hour and then heated at 80 °C in an oven for 4 h. Once the PDMS microchip was removed from the "mold", each of the 18 mini-vial was punched to have two inlets for sample injection using a flat-end needle (1 mm in diameter, as shown in the main text, Scheme 1B).

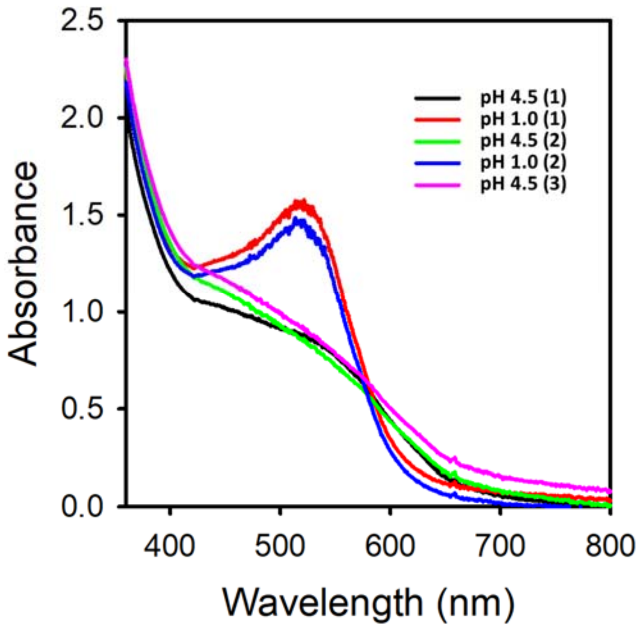

Figure S1. Reversibility test of the pH dependent structural change of anthocyanidins.

UV-Vis absorption spectra of the “fermented Rosso” sample upon changing the pH from 1.0 to 4.5 and vice versa. The numbers in brackets on the legend indicates the number of cycles. The result is in agreement with previous reports; as example, see Tang, B.; He, Y.; Liu, J.; Zhang, J.; Li, J.; Zhou, J.; Ye, Y.; Wang, J.; Wang, X. Kinetic Investigation into pH-dependent Color of Anthocyanin and Its Sensing Performance. *Dyes Pigm.* **2019**, *170*, 107643.

Table S1. Three replicate UV-Vis measurements (absorbance at 520 nm) of Cy3G standard solutions.

The average absorbance (Avg) and standard deviation (SD) were determined accordingly and shown in Figure 3a of the main text.

[Cy3G] (μg/mL)	Abs (520 nm)									
	1		2		3		Avg		SD	
	pH 1.0	pH 4.5	pH 1.0	pH 4.5	pH 1.0	pH 4.5	pH 1.0	pH 4.5	pH 1.0	pH 4.5
**28.0**	1.045	0.107	1.006	0.098	1.008	0.093	1.020	0.099	0.022	0.007
**17.5**	0.706	0.072	0.696	0.039	0.663	0.060	0.688	0.057	0.023	0.017
**10.9**	0.466	0.041	0.424	0.039	0.439	0.045	0.443	0.042	0.021	0.003
**6.84**	0.286	0.032	0.281	0.019	0.274	0.034	0.280	0.028	0.006	0.008
**4.27**	0.144	0.031	0.151	0.016	0.143	0.015	0.146	0.021	0.004	0.009
**1.67**	0.074	0.013	0.105	0.013	0.069	0.012	0.083	0.013	0.020	0.001
**1.04**	0.070	0.021	0.056	0.006	0.044	0.006	0.057	0.011	0.013	0.009
**0.65**	0.034	0.018	0.044	0.012	0.031	0.006	0.036	0.012	0.007	0.006
**0.41**	0.032	0.013	0.041	0.000	0.019	0.010	0.031	0.008	0.011	0.007

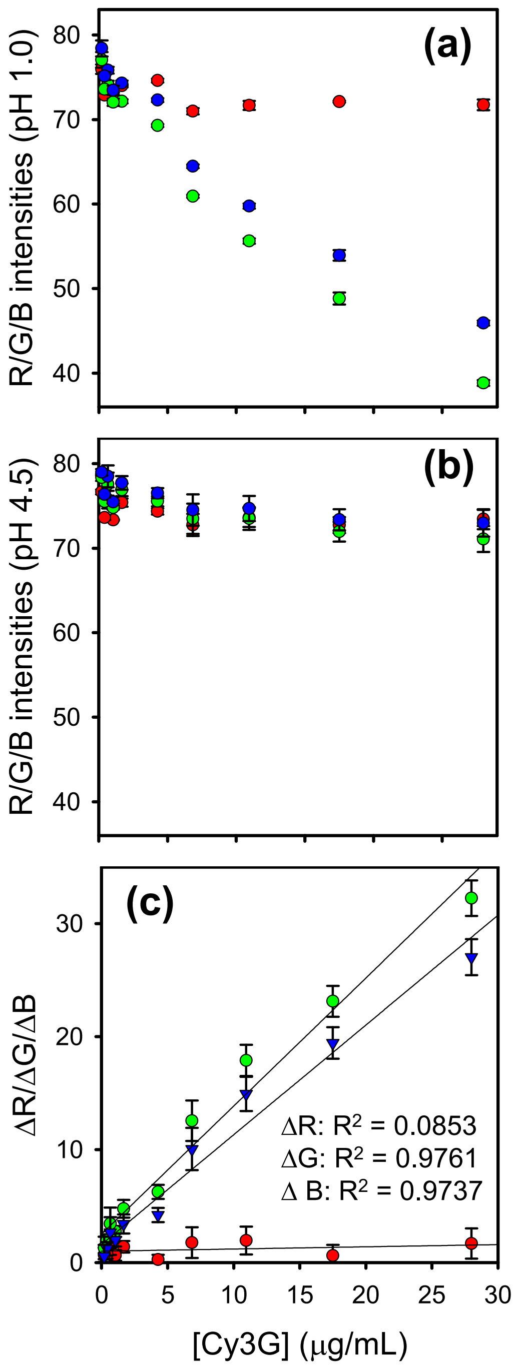

Figure S2. R, G, and B intensities of Cy3G standard solutions at different concentrations.

(a) pH 1.0; (b) pH 4.5; the values were obtained from the image shown in Figure 4 of the main text. (C) ΔR, ΔG, and ΔB (differences between the values at pH 1.0 and pH 4.5) vs. the Cy3G concentration, where the solid lines show the best linear fits to the experimental data. All data points are color-coded correspondingly, i.e., red for R, blue for B, and green for G. The linearity of these fits is not comparable with that of Figure 5B (ΔR/RGB vs. [Cy3G]; $R^2 = 0.999$).

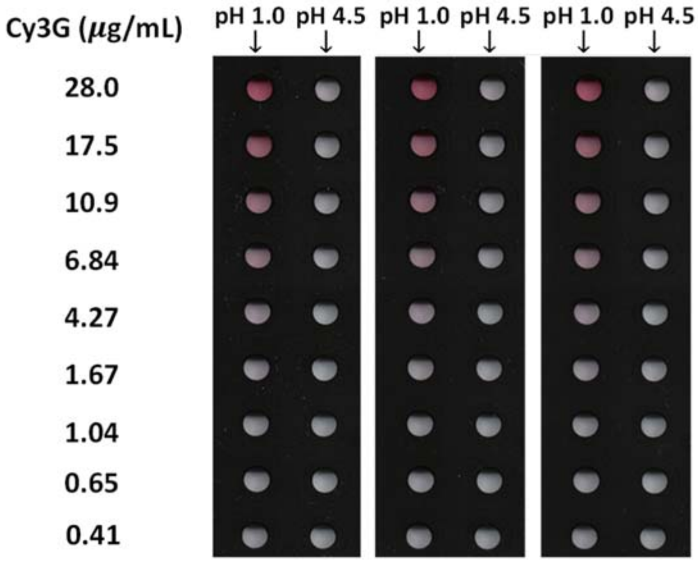

Figure S3. Three replicate experiments to determine the colorimetric response of Cy3G standard solutions with the microchip device.

For each trial, Cy3G standard solutions of different concentration were adjusted to two different pH values, followed by scanning with the portable scanner, sending photos to a smartphone, and analyzing using the Color Grab app. Once the RGB values for each mini-vial were obtained from the app (Figure 5a), a calibration curve to correlate ΔR/RGB and the concentration of Cy3G was then established (Figure 5b); these replicates were to determine the standard deviations shown as error bars (see main text for details).

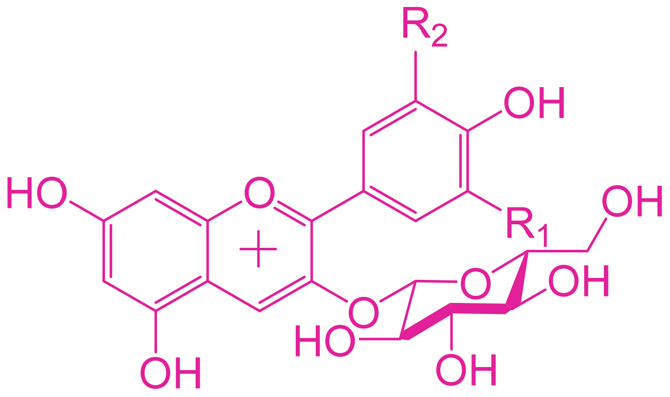

	Compound name	R_1	R_2	Approximate abundance %
Anthocyanin	Pelargonidin-3-*O*-glucoside	H	H	12
	Cyanidin-3-*O*-glucoside	OH	H	50
	Delphinidin-3-*O*-glucoside	OH	OH	12
	Peonidin-3-*O*-glucoside	OCH_3	H	12
	Petunidin-3-*O*-glucoside	OCH_3	OH	7
	Malvidin-3-*O*-glucoside	OCH_3	OCH_3	7

Table S2. Six naturally occurring anthocyanidins and their corresponding abundance.

See, Olivas-Aguirre, F.; Rodrigo-García, J.; Martínez-Ruiz, N.; Cárdenas-Robles, A.; Mendoza-Díaz, S.; Álvarez-Parrilla, E.; González-Aguilar, G.; de la Rosa, L.; Ramos-Jiménez, A.; Wall-Medrano, A. Cyanidin-3-O-Glucoside: Physical-Chemistry, Foodomics and Health Effects. *Molecules* **2016**, *21*, 1264.

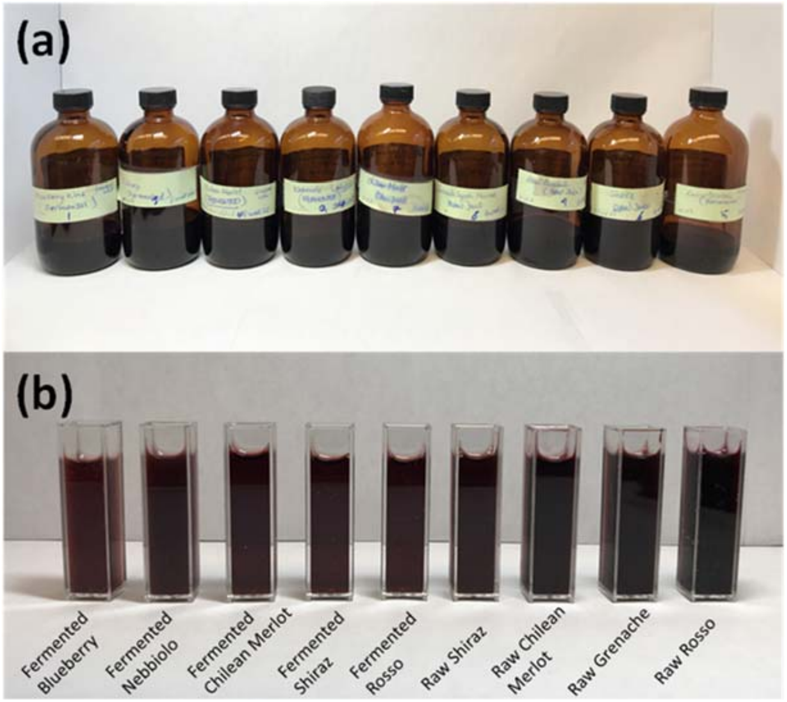

Figure S4. Tested beverage samples and their natural colors.

(a) Picture of the fermented wine and raw juice samples provided by the Bayou Brewing Club, which is a sub-division of Laca Biotech Inc., and (b) the natural colors of each beverage as received. Among the nine sample beverages, the "raw" ones are the original grape juice; the "fermented" ones are the wines made from their corresponding raw juices. We can see that the raw juices are generally darker than the fermented ones, indicative of the higher abundance of total anthocyanidins.

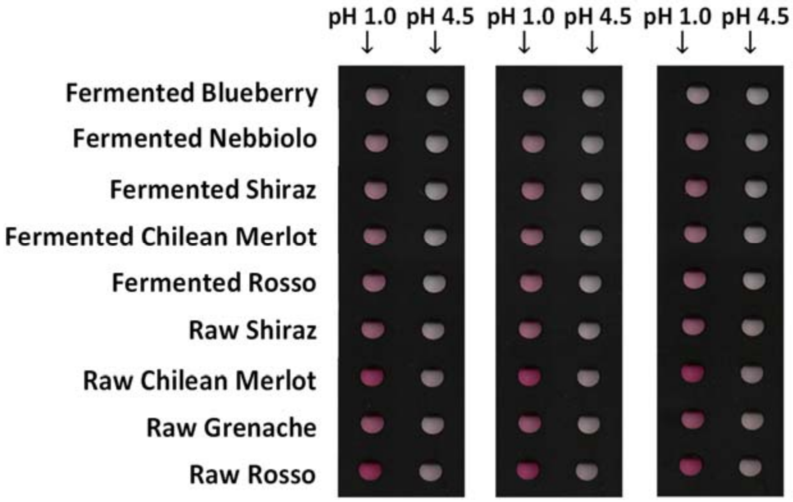

Figure S5. Three replicate experiments for determining the concentrations of Cy3G in different beverage samples with the microchip device.

According to the established calibration curve with standard Cy3G solutions (Figure 5), the concentration of Cy3G in each sample tested were determined; the standard deviations were obtained from these three repeated trials, as presented in Figure 6 (see main text for details).
